# Fine-Root Turnover, Litterfall, and Soil Microbial Community of Three Mixed Coniferous–Deciduous Forests Dominated by Korean Pine (*Pinus koraiensis*) Along a Latitudinal Gradient

**DOI:** 10.3389/fpls.2019.01298

**Published:** 2019-10-24

**Authors:** Lu Liu, Fan Yang, YuJue Wang, Xing Shen, Ivan A. Janssens, Bertrand Guenet, Chunwang Xiao

**Affiliations:** ^1^College of Life and Environmental Sciences, Minzu University of China, Beijing, China; ^2^Hulun Lake Reserve Grassland Ecology Research Station, Minzu University of China, Beijing, China; ^3^State Key Laboratory of Vegetation and Environmental Change, Institute of Botany, Chinese Academy of Sciences, Beijing, China; ^4^Department of Biology, Research Group of Plants and Ecosystems, University of Antwerp, Wilrijk, Belgium; ^5^Laboratoire des Sciences du Climat et de l’Environnement, LSCE/IPSL, CEA-CNRS-UVSQ, Université Paris-Saclay, Gif-sur-Yvette, France

**Keywords:** fine roots, litter decomposition, soil organic carbon, Korean pine, latitudinal gradient

## Abstract

Carbon dynamics in forests and in particular in soils are of primary importance in the context of climate change. A better understanding of the drivers controlling carbon storage is needed to improve climate mitigation strategies. Carbon storage is the result of a balance between inputs and outputs. Carbon inputs in the soil come from plant residues and root exudates, which are further transformed by microorganisms and stored in the long term. Here, we measured litter and fine-root production in three mixed forests dominated by *Pinus koraiensis* along a latitudinal gradient and performed a litterbag experiment to better understand the driving factors of decomposition. We found that over the three sites litter production was controlled by climatic factors, soil properties, and forest stand characteristics, whereas decay rates were mainly controlled by microbial community structure and soil stoichiometry. For fine roots, production differed among sites, and higher production was consistently observed in the top soil layers compared to deep soil, although the root distribution along the soil profile differed among sites. Fine-root decay rates were mainly controlled by fine-root stoichiometric characteristics. This article emphasizes the complexity of fine-root dynamics even for a single species. Environmental drivers impact on both production and decay, and we suggest performing manipulative field experiments to better identify the mechanisms involved in soil carbon cycling.

## Introduction

The interest in soil organic carbon (SOC) has increased in recent decades (Feller and Bernoux, 2008). Soils contain the largest stock of organic carbon, exceeding the amount of carbon stored in the atmosphere and in plant biomass (Jobbágy and Jackson, 2000; Scharlemann et al., 2014). As a consequence, even a small change in soil carbon storage may significantly affect the atmospheric concentrations of greenhouse gases and therefore the climate (Rustad et al., 2000). Thus, SOC is considered of major importance in the global carbon cycle (Schmidt et al., 2011), and a better understanding of the factors controlling SOC dynamics is urgently needed to mitigate climate change (Luo et al., 2016; Dignac et al., 2017).

The SOC stocks are controlled by balance between the outputs (i.e., heterotrophic respiration and dissolved organic carbon lateral transport and erosion) and the inputs from primary production and sometimes deposition of eroded materials (Guo et al., 2005). The OC inputs into the soils can originate from aboveground litter entering the soil or come from root exudates (Rasse et al., 2005). Root production depends on local conditions, such as soil water content (SWC) and nutrient availability, plant species and ecological interactions with other species (Gill and Jackson, 2000; Finér et al., 2011; Keel et al., 2012). However, inputs into the soil depend also on root mortality, which is controlled by plant phenology and external drivers such as herbivory, fire, or environmental conditions (Riley et al., 2010). Finally, to be stored in the long term, dead plant material must be incorporated into the microbial biomass (Kallenbach et al., 2016). Microbial uptake and biotransformation are controlled by the structure of the microbial community as well as environmental drivers (Kaiser et al., 2014). Disentangling the effects of plant characteristics (e.g., litter quality) from the effects of environmental conditions (e.g., soil characteristics) is very complex. Therefore, major efforts are still needed toward a better understanding of root dynamics and in particular of the controlling factors of dead root decomposition. However, fine-root and litter turnover, decomposition of fine root and litter, and characteristics of soil microbial biomass have been rarely reported simultaneously in past studies. So, it might be important to find out its underlying mechanism.

In this study, we focused on factors driving the dynamics of root and litterfall at three different mixed forest sites in Northeast China dominated by *Pinus koraiensis* Sieb. et Zucc. (Korean pine) along a latitudinal gradient. These three sites have different vegetation composition and soil characteristics. Our major goals were to determine the seasonal dynamics of fine-root biomass and litterfall and fine-root and litter turnover rate and to understand the major drivers. We assumed that (1) fine-root and leaf litter decomposition rates were related to soil microbial properties in the Korean pine forests from different sites, but (2) those relationships regulating the fine-root and litter dynamics vary along latitudinal and gradients.

## Materials and Methods

### Site Description

Three mixed coniferous–deciduous forests dominated by the Korean pine along a latitudinal gradient in Northeast China were assessed in this study: (1) Kuandian (40°91′N, 124°79′E, 930 m a.s.l.), (2) Dongsheng (44°41′N, 128°12′E, 804 m a.s.l.), (3) Shengshan (49°48′N, 126°78′E, 504 m a.s.l.). This region has a temperate continental climate with long cold winters but warm summers. The mean annual temperature and mean annual precipitation in Kuandian, Dongsheng, and Shengshan are 4.92°C and 1,082 mm, 0°C and 717 mm, and −1.54°C and 561 mm, respectively. Soils at the three sites are classified as Eutric cambisols (FAO-WRB (ISSS-ISRIC-FAO-UNESCO), 1998).

Three 20 × 20-m plots of mixed coniferous–deciduous forests dominated by Korean pine at each site were selected for this study. In Kuandian, the forest was dominated by *P. koraiensis* Sieb. et Zucc.(Korean pine), admixed with *Abies nephrolepis* (Trautv.) Maxim., *Padus maackii* (Rupr.) Kom., *Betula costata* Trautv., *Acer pseudosieboldianum* (Pax) Komarov, *Tilia amurensis* Rupr., *Quercus mongolica* Fisch. ex Ledeb., *Ulmus laciniata* (Trautv.) Mayr. In Dongsheng, the forest was dominated by Korean pine, admixed *Abies holophylla* Maxim., *Acer mono* Maxim., *Betula platyphylla* Suk., *Quercus mongolica* Fisch. ex Ledeb., and *T. amurensis* Rupr. Finally, in Shengshan, the forest was dominated by *P. koraiensis* Sieb. et Zucc., admixed with *T. amurensis* Rupr., *U. laciniata* (Trautv.) Mayr., and *A. mono* Maxim. Tree density, stand basal area, and Korean pine basal area of the three forests are shown in **Table 1**.

**Table 1:** The community and soil properties of three mixed coniferous–deciduous forests dominated by Korean pine and located in Kuandian, Dongsheng, and Shengshan. Values represent mean ± standard error (n = 3). Different letters within a soil layer are significantly different (*P* < 0.05) according to the Duncan *post hoc* test.

	Stand density (stem ha^−1^)	Stand basal area (m^2^ ha^−1^)	Korean pine basal area (m^2^ ha^−1^)	Soil water content (%)	Soil bulk density (g cm^−3^)	pH	Soil organic carbon (g kg^−1^)	Soil total nitrogen (g kg^−1^)	Soil total phosphorus (g kg^−1^)	C:N	C:P	N:P
Kuandian	867 ± 203a	35.5 ± 0.5b	12.2 ± 2.4b	69.07 ± 1.77a	1.08 ± 0.01a	4.87 ± 0.13a	66.4 ± 3.2ab	4.8 ± 0.1a	0.75 ± 0.04a	13.8 ± 0.3b	88.6 ± 2.0b	6.5 ± 0.2a
Dongsheng	878 ± 121a	65.6 ± 10.7a	20.6 ± 5.0a	49.6 ± 2.71b	1.1 ± 0.02a	4.97 ± 0.09a	74.1 ± 3.4a	5.1 ± 0.1a	0.79 ± 0.03a	14.7 ± 0.4b	93.8 ± 2.1b	6.4 ± 0.1a
Shengshan	692 ± 224a	28.6 ± 2.4b	21.8 ± 0.9a	35.8 ± 1.63c	1.07 ± 0.01a	4.90 ± 0.06a	57.8 ± 3.0b	3.1 ± 0.1b	0.53 ± 0.02b	18.6 ± 0.5a	109.7 ± 2.6a	5.9 ± 0.1a

### Soil Properties

Soil samples (0–20 cm) for measuring gravimetric SWC were collected from five randomly placed cores (3-cm diameter) in each plot in May, June, July, August, September, and October 2015, and SWC was measured by oven-drying samples at 105°C for 24 h. Soil bulk density was determined by weighing oven-dried samples of known volume by using a cutting ring (volume 100 cm3, inner diameter 5 cm). Soil samples (0–20 cm) for measuring physical and chemical properties were collected from five randomly placed cores (3-cm diameter) in each plot in July 2015. The five replicates in each plot were pooled and mixed to get one composite sample. Composite soil samples were air dried, passed through a 2-mm sieve, and manually cleaned of any visible plant tissues for laboratory analysis. Soil organic carbon content and soil total nitrogen content were determined by an element analyzer (Vario EL III), and soil total phosphorous content (Moty et al., 2016) was analyzed by using the molybdenum blue colorimetric method with a UV/visible spectrophotometer after H2SO4-H2O2 digestion (UV-2550; Shimadzu, Kyoto, Japan).

### Fine-Root Biomass, Production, and Turnover Rate

The core sampling method (Roberts, 1976) was used to measure fine-root (< 2 mm) biomass in each plot down to 40-cm soil depth in May, July, and September 2015. At each sampling date, soil samples (0–10, 10–20, and 20–40 cm) were collected from five randomly located cores (8 cm in diameter and 10 cm in length) at each plot. Fine roots were manually removed from the soil samples and washed. These separated fine roots were further classified into live and dead fine roots by the following distinct criteria. Live fine roots were characterized as roots with a varying degree of brownish tissues, often well branched, with light and turgid root tips, and with white to slightly brown and elastic stele (Vogt and Persson, 1990; Persson and Stadenberg, 2010; Yang et al., 2010). Dry biomass was determined after oven drying at 65°C for 2 days.

A modified in-growth core technique (Lund et al., 1970) was used to determine fine-root (< 2 mm) production. Five holes were created in each plot and were refilled with native soil without any root biomass, and their boundaries were marked with sticks in October 2014. These in-growth cores were harvested at the end of October 2015. Soil samples from different depths (0–10, 10–20, and 20–40 cm) for each in-growth core were labeled, and fine-root biomass was subsequently estimated using exactly the same procedures as described above. Total fine-root production was estimated as the sum of live and dead roots present in the in-growth core at the end of October 2015.

Fine-root turnover rate is defined here as the ratio of the total amount of fine root produced over the mean standing biomass of fine roots (Aber et al., 1985). Mean fine-root biomass was estimated as the average of live root biomass in May, July, and September 2015.

### Forest Floor Mass, Litter Fall, and Litter Turnover

Forest floor mass was collected in October 2014 and every month from May to October 2015 by placing a 0.5 × 0.5-m quadrant at five randomly located places in each plot. Collected forest floor mass was further sorted into woody and nonwoody fractions. Litterfall samples were trapped and collected using five randomly located 0.5 × 0.5-m rectangular baskets in each plot. Samples were frequently collected from October 2014 to October 2015 and sorted into woody and nonwoody fractions. Dry litter mass was determined after oven drying at 65°C for 2 to 3 days.

The turnover rate of litter was calculated as the ratio of the annual litter production over the mean annual floor mass according to (Rochow, 1974). Mean annual floor mass was estimated as the average of floor mass between October 2014 and October 2015.

### Soil Microbial Properties

Five 0- to 20-cm soil core samples (3-cm diameter) per plot were randomly collected for determination of soil microbial properties in July 2015. Each sample was labeled and then stored at 2°C in a cooler before transporting to the laboratory. In the laboratory, the fresh samples were passed through a 2-mm sieve and manually cleaned of any visible plant tissues. Soil microbial biomass carbon (SMBC) and nitrogen (SMBN) were measured by the chloroform fumigation–extraction method (Vance et al., 1987). Briefly, after adjusting to approximately 60% of water-holding capacity, the fresh soil samples were incubated for 1 week in the dark at 25°C. Twenty grams (dry weight equivalent) of fumigated and nonfumigated soil samples were extracted with 0.5 M K_2_SO_4_. Extracts were filtered through 0.45-µm filters and frozen at −20°C before analysis of extractable carbon by dichromate digestion as described by Lovell et al. (1995). SMBC and SMBN were calculated as the difference in extractable organic C and inorganic N contents between the fumigated and the nonfumigated samples using conversion factors (kec and ken) of 0.38 and 0.45 (Lovell et al., 1995), respectively.

The soil microbial community was characterized using phospholipid fatty acids (PLFAs) analysis as described by Bossio and Scow (1998). The separation and identification of extracted PLFAs were carried out according to the standard protocol of the Sherlock Microbial Identification System V_4.5_ (MIDI) and a Gas Chromatograph (Agilent 6850, USA). The abundance of individual fatty acids was determined as relative nanomoles per gram of dry soil, and standard nomenclature was used. The fatty acids i15:0, a15:0, 15:0, i16:0, 16:1ω7c, 16:1ω5c, i17:0, a17:0, 17:0cy, and 19:0cy were chosen to represent the PLFAs of the bacterial group, and fungi were considered to be represented by the PLFAs 18:2ω6c and 18:1ω9c (Frostegård and Bååth, 1996; Bossio and Scow, 1998). All of the PLFAs mentioned above were used to calculate the total PLFAs of soil microbial community. The ratio of fungal to bacterial PLFAs was also included in the data analysis. This ratio has often been used as an indicator of changes in the soil microbial community structure (Bardgett et al., 1999).

### Decomposition of Leaf Litter and Fine Roots

Decomposition rates of leaf litter and fine root were determined using the nylon bag (or litterbag) method (Arunachalam et al., 1996). Recently fallen leaves and fine roots from the 0- to 10-cm mineral soil layer were collected from each plot.

Each nylon bag had a dimension of 15 × 15 cm and a mesh of 1 mm. In each plot, 25 nylon bags containing 3 g of air-dried leaves or fine roots were placed on the forest floor and at the10-cm soil depth, respectively, in October 2014. These nylon bags were sampled after 0, 218, 263, 307, and 365 days in Kuandian; 0, 220, 264, 308, and 365 days in Dongsheng; and 0, 221, 266, 309, and 365 days in Shengshan, respectively. Five nylon bags were collected at each sampling date. Mass of leaf litter and fine roots in each nylon bag was determined after oven drying at 65°C for 2 to 3 days. In addition, we also determined total C, N, and P of leaf litter and fine-root material (**Table 2**). Initial OC, total N, and P content of each litter category were measured using the same methods as for soil.

**Table 2:** The chemical contents of leaf and fine-root litter of mixed coniferous–deciduous forests dominated by Korean pine in Kuandian, Dongsheng, and Shengshan. Values represent mean ± standard error (n = 3). Different letters within a soil layer are significantly different (*P* < 0.05) according to the Duncan *post hoc* test.

		Kuandian	Dongsheng	Shengshan
**Leaf litter**	C	504.7 ± 4.2b	513.2 ± 7.3b	533.5 ± 3.3a
	N	14.9 ± 0.1a	13.7 ± 0.3b	12.5 ± 0.2c
	P	1.1 ± 0.01a	1.1 ± 0.02a	0.98 ± 0.02b
	C:N	34.0 ± 0.2b	37.5 ± 0.6c	42.7 ± 0.6a
	N:P	13.0 ± 0.12a	12.7 ± 0.02a	12.7 ± 0.11a
	C:P	441.5 ± 2.1b	475.5 ± 8.2c	544.9 ± 11.5a

**Fine-root litter**	C	472.4 ± 3.1b	482.3 ± 3.6b	497.9 ± 2.1a
	N	14.2 ± 0.4a	12.2 ± 0.2b	11.2 ± 0.4b
	P	1.2 ± 0.03a	1.2 ± 0.01a	1.0 ± 0.02b
	C:N	33.2 ± 0.8b	39.6 ± 0.5c	44.7 ± 1.5a
	N:P	11.6 ± 0.1a	10.6 ± 0.2b	10.8 ± 0.5ab
	C:P	386.5 ± 7.6b	419.5 ± 3.0c	480.6 ± 7.9a

### Statistical Analysis

Data management and statistical analyses were performed using SPSS software (SPSS, Chicago, IL). One-way analysis of variance (ANOVA) was used to test for significant differences of community and soil properties, initial chemical content of leaf litter and fine root, and the annual decay constant (*k*). Two-way ANOVA was used to test the effects of sampling position and soil layer on fine-root production, and two-way ANOVA was used to test the effects of sampling position and sampling date on litter fall and forest floor mass. Three-way ANOVA was used to test the effects of sampling position, soil layer, and sampling time and all their interactions on fine-root biomass. Multiple comparisons were also performed to permit separation of effect means using Duncan *post hoc* test at a significance level of *P* < 0.05. A stepwise regression was used to measure the relationships of the annual decay constant (*k*) of leaf litter or fine-root litter to soil microbial community and soil properties, initial chemical content of leaf litter and fine root, fine-root biomass, production and turnover rate, litter fall and forest floor mass, and soil microbial properties. This stepwise regression was performed on the entire dataset without distinction per site. Annual decay constant was calculated using Equation 1 (Olson, 1963):

(1) $$Y_{t}=\;Y_{0}\times \;e^{\text{​​}-}{}^{\text{​​}k\text{​​}t}$$

with *t* being the time in years; *Y*_0_, the weight of litter at the beginning of the experiment; *Y* *_t_*, the weight of remaining litter at certain time (*t*); and *k*, the decay constant.

## Results

### Forest Floor Mass and Litter Fall

**Figure 1:**
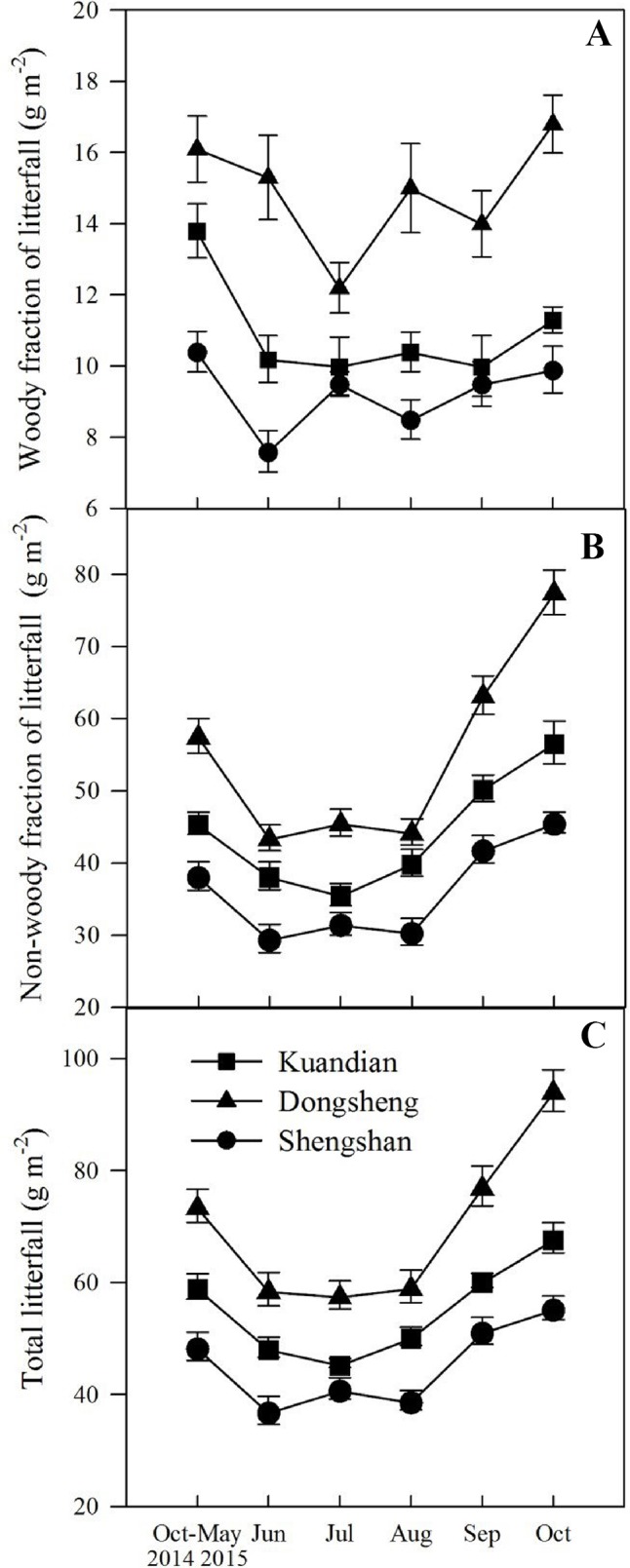
Seasonal changes in woody **(A)** and nonwoody **(B)** fractions of litterfall and total litterfall **(C)** of three mixed coniferous–deciduous forests dominated by Korean pine and located in Kuandian, Dongsheng, and Shengshan from October 2014 to October 2015. Vertical bars indicate standard errors of means (n = 3).

Total litter fall was site dependent (**Table 3**, *P* < 0.001), with the highest litter production in Donsheng, followed by Kuandian and Shengshan (**Figure 1**). For all sites, litter production varied seasonally (**Table 3**, *P* < 0.001), and a significant interaction between site and time was observed (**Table 3**, *P* < 0.001). In particular, the peak in litter fall in September was higher for Donsheng than for the other sites. The woody fraction of the litter fall followed the same seasonal pattern as total litter fall, with a site and a time effect, but no interaction between both (**Table 3**). For the nonwoody fraction, a significant interaction between site and time was also detected.

**Table 3:** Results (*F* values) of three-way ANOVA on the effects of site, soil layer (SL), sampling time (ST), and all their interactions on fine-root biomass and production, woody and nonwoody fraction of litter fall and total litter fall, and woody fraction and nonwoody fraction of forest floor mass and total forest floor mass.

Sources	Fine-root biomass	Fine-root production	Woody fraction of litter fall	Nonwoody fraction of litter fall	Total litter fall	Woody fraction of forest floor mass	Nonwoody fraction of forest floor mass	Total forest floor mass
Site	174.30***	127.01***	86***	130.38***	154.06***	29.67***	913.49***	466.81***
ST	12.79***		6.23***	62.06***	50.02***	0.76	7.05***	4.39**
SL	174.30***	505.28***						
Site * ST	0.35		1.88	3.622**	2.94**	0.10	0.34	0.15
ST * SL	3.46*							
Site * SL	38.47***	16.26***						
Site * ST * SL	0.11							

*, **, and *** represent significance at P < 0.05, P < 0.01, and P < 0.001.

**Figure 2:**
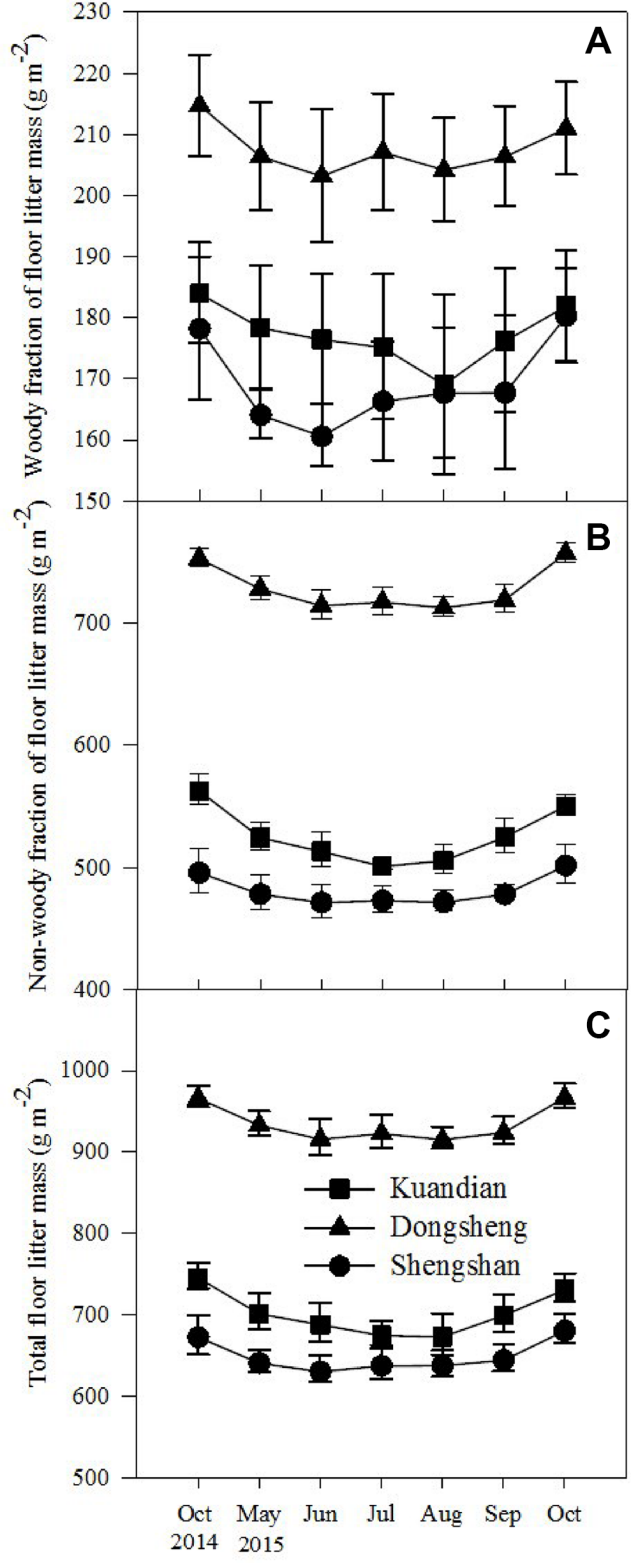
Seasonal changes in woody fraction **(A)** and nonwoody fraction **(B)** of forest floor mass and total forest floor mass **(C)** of three mixed coniferous–deciduous forests from October 2014 to October 2015. Vertical bars indicate standard errors of means (n = 3).

Total floor mass showed a similar pattern to that of litterfall, except that no interaction between site and time was observed (**Table 3**). The total floor mass was higher for Donsheng followed by Kuandian and Shengshan (**Figure 2**), and following the litter fall peak, also total floor litter mass was higher in September and October. For the woody fraction, only a site effect was detected (**Table 3**, *P* < 0.001), and no significantly temporal variation was observed. For the nonwoody fraction, a site effect together with a time effect was detected (for both **Table 3**, *P* < 0.001).

### Fine-Root Biomass, Production, and Turnover Rate

**Figure 3:**
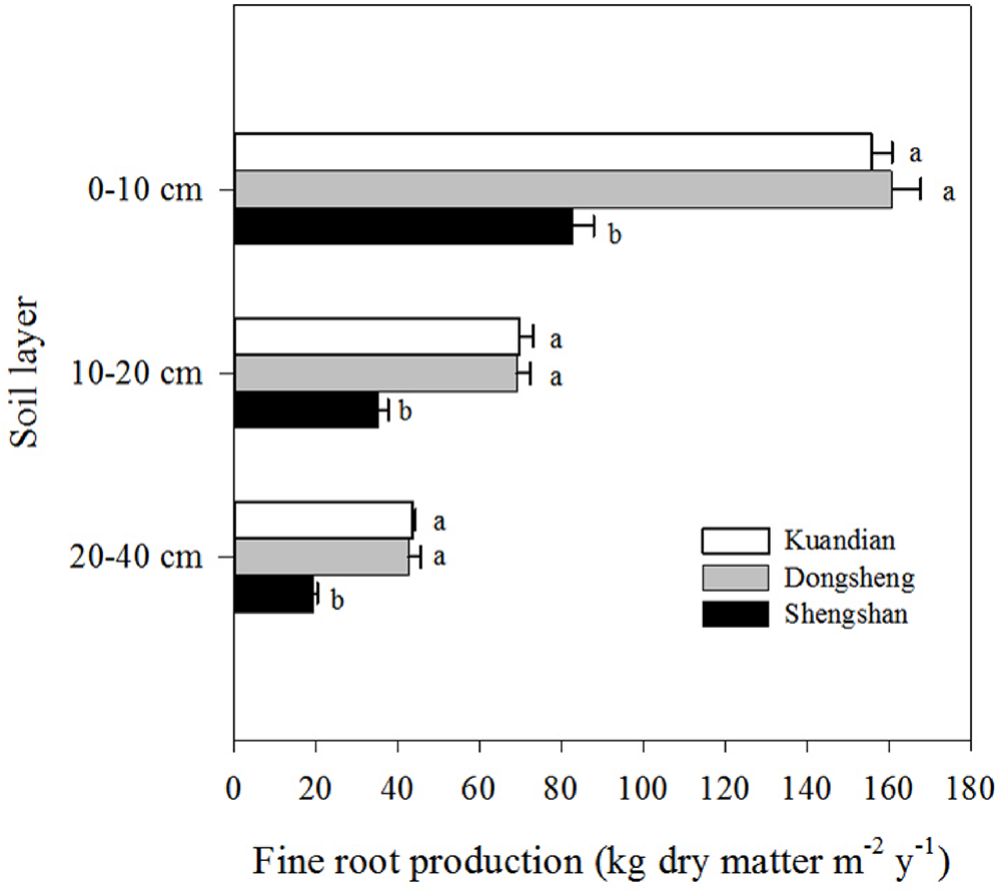
Vertical distribution of fine-root production of three mixed coniferous–deciduous forests. Horizontal bars indicate standard errors of means (n = 3). Different letters within a soil layer indicate significant difference (*P* < 0.05) according to the Duncan *post hoc* test.

**Figure 4:**
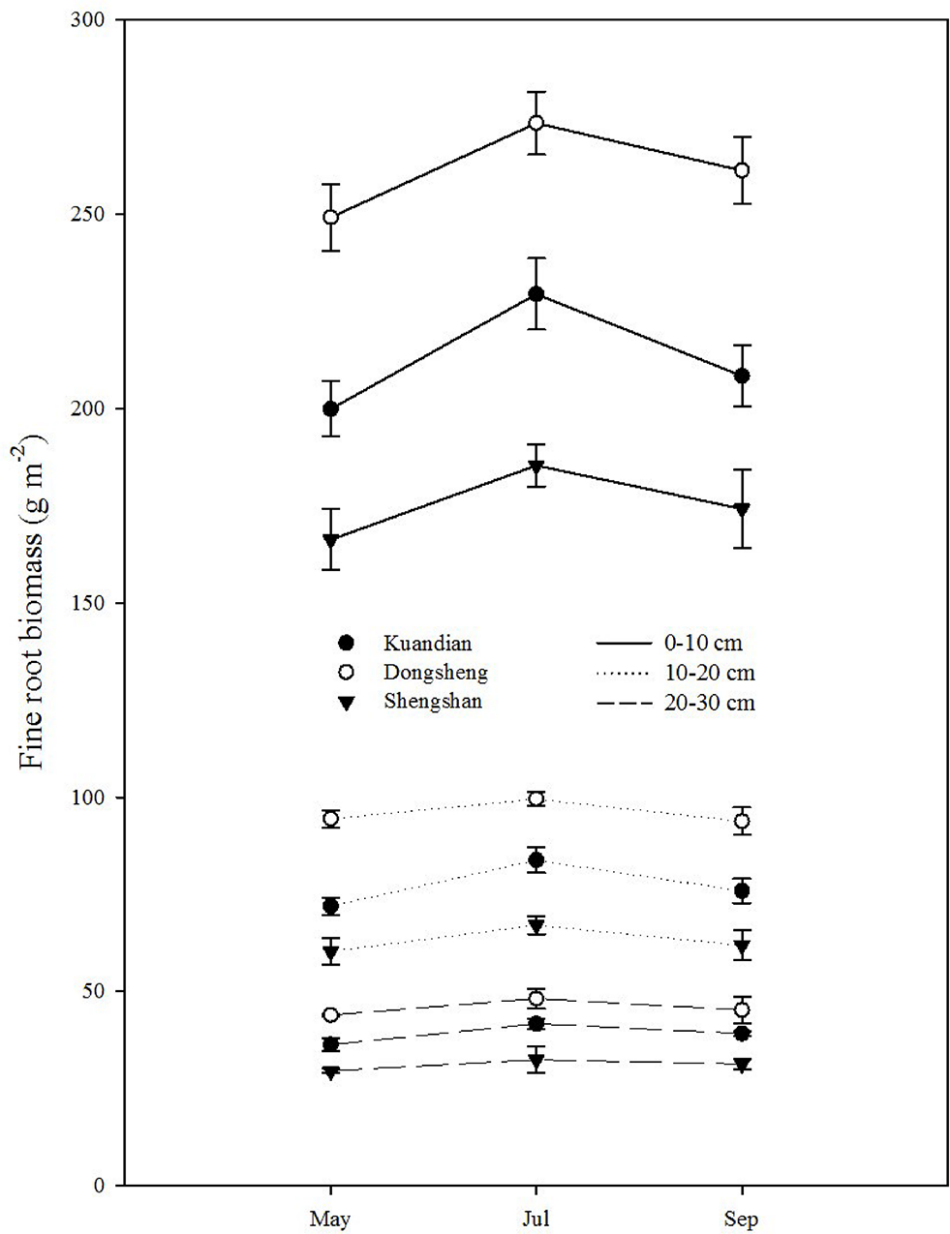
Vertical distribution of fine-root biomass of three mixed coniferous–deciduous forests in May, July, and September 2015. Vertical bars indicate standard errors of means (n = 3).

Fine-root biomass production was site dependent (**Table 3**, *P* < 0.001), with higher production at Donsheng, followed by Kuandian and Shengshan (**Figure 3**). All sites presented higher fine-root production in top soil compared to deeper soil layers (**Figure 3**，**Table 3**). This vertical pattern was site dependent, as suggested by the significant interaction between site and soil layers presented in **Table 3**. Nevertheless, this effect was not quantitatively important (**Figure 3**). Fine-root biomass followed a similar pattern (**Figure 4**), with higher fine-root biomass in the top soil layers than in the deep soil layers. We also observed a site effect with higher biomass for Donsheng followed by Kuandian and Shengshan. Fine-root biomass also varied with time (**Table 3**, *P* < 0.001), with higher biomass in July compared to the other periods. For fine-root biomass, the significant interaction between time and soil layers (**Table 3**, *P* < 0.05) suggests that the increase observed in July was mainly due to an increase in the top layers (**Figure 4**). Finally, the significant interaction between site and soil layers (**Table 3**, *P* < 0.001) suggests that the vertical root biomass distribution was site dependent. Indeed, the relative proportion of fine-root biomass was highest in the top layer; this vertical difference was larger at Shengshan than at the other sites.

### Soil Microbial Properties

**Figure 5:**
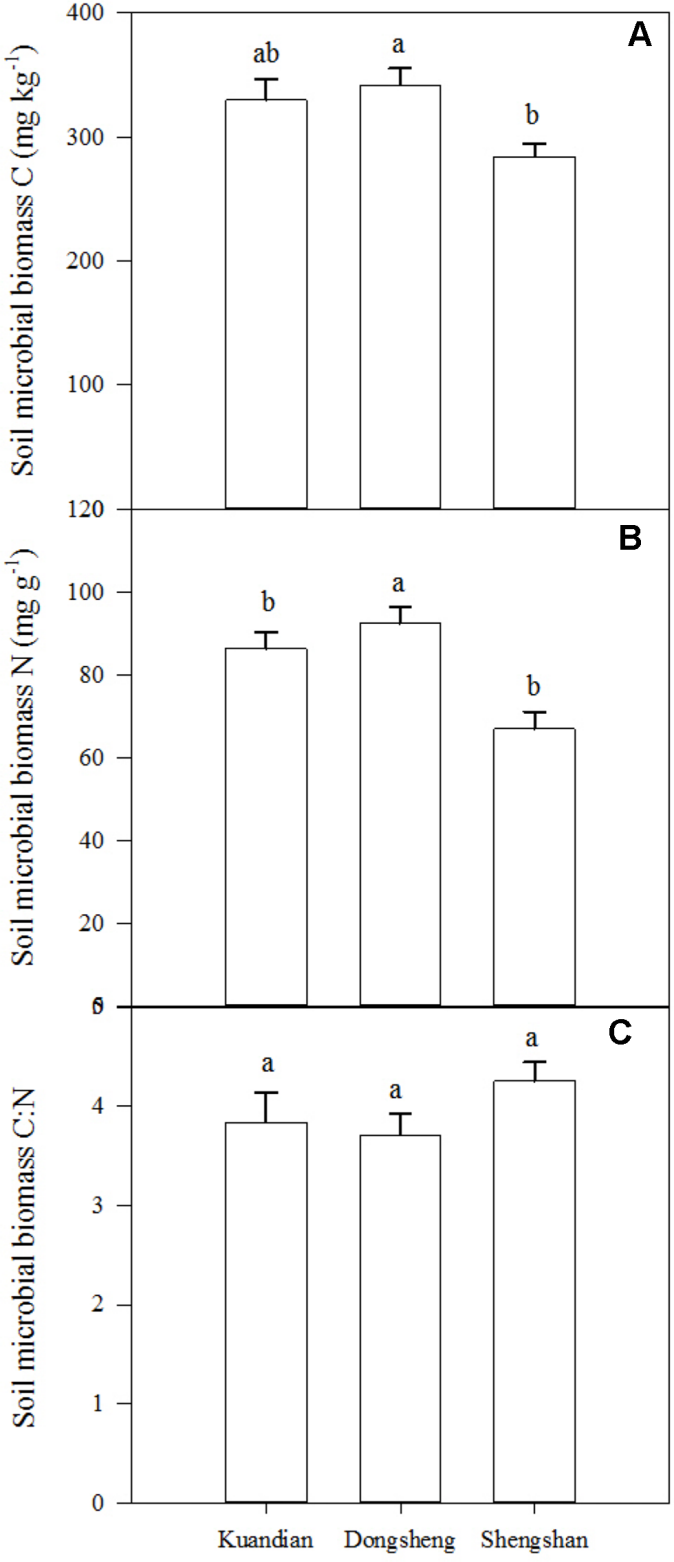
Soil microbial biomass C **(A)**, N **(B)**, and C: N **(C)** ratio of three mixed coniferous–deciduous forests in the 0- to 20-cm-depth increment. Vertical bars indicate standard errors of means (n = 3). Different letters indicate significant difference (*P* < 0.05) according to the Duncan *post hoc* test.

**Figure 6:**
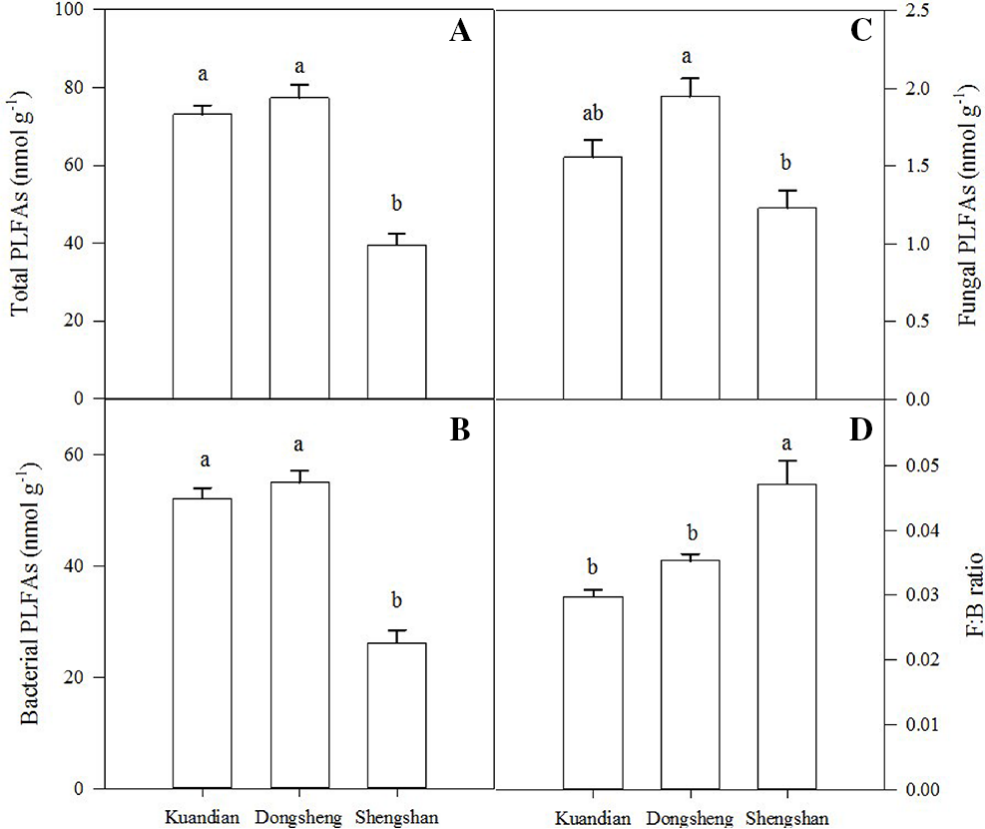
Total PLFAs **(A)**, bacterial PLFAs **(B)**, fungal PLFAs **(C)**, and F:B ratio **(D)** of mixed coniferous–deciduous forests dominated by Korean pine in Kuandian, Dongsheng, and Shengshan. Vertical bars indicate standard errors of means (n = 3). Different letters within a soil layer are significantly different (*P* < 0.05) according to the Duncan *post hoc* test.

Soil microbial biomass C was similar in Donsheng and Kuandian, but lower in Shengshan (**Figure 5**). For soil microbial N, a higher stock was observed in Donsheng, followed by Kuandian and Shengshan. Nevertheless, the microbial C:N ratio was similar at all sites, being close to 4. For all sites, the microbial community was dominated by bacteria, as suggested by the PLFA analysis presented in **Figure 6**. Indeed, the ratio of fungi to bacteria (F:B ratio) was low: between 0.0298 ± 0.0011 for Kuandian and 0.0471 ± 0.0035 for Shengshan. Some differences among sites were observed. In particular, in agreement with the other proxy for microbial biomass, soil microbial C, also the total PLFA was significantly lower at Shengshan compared to the other sites. Also, fungal biomass was higher at this site, explaining its significantly higher F:B ratio.

### Leaf Litter and Fine-Root Decomposition

**Figure 7:**
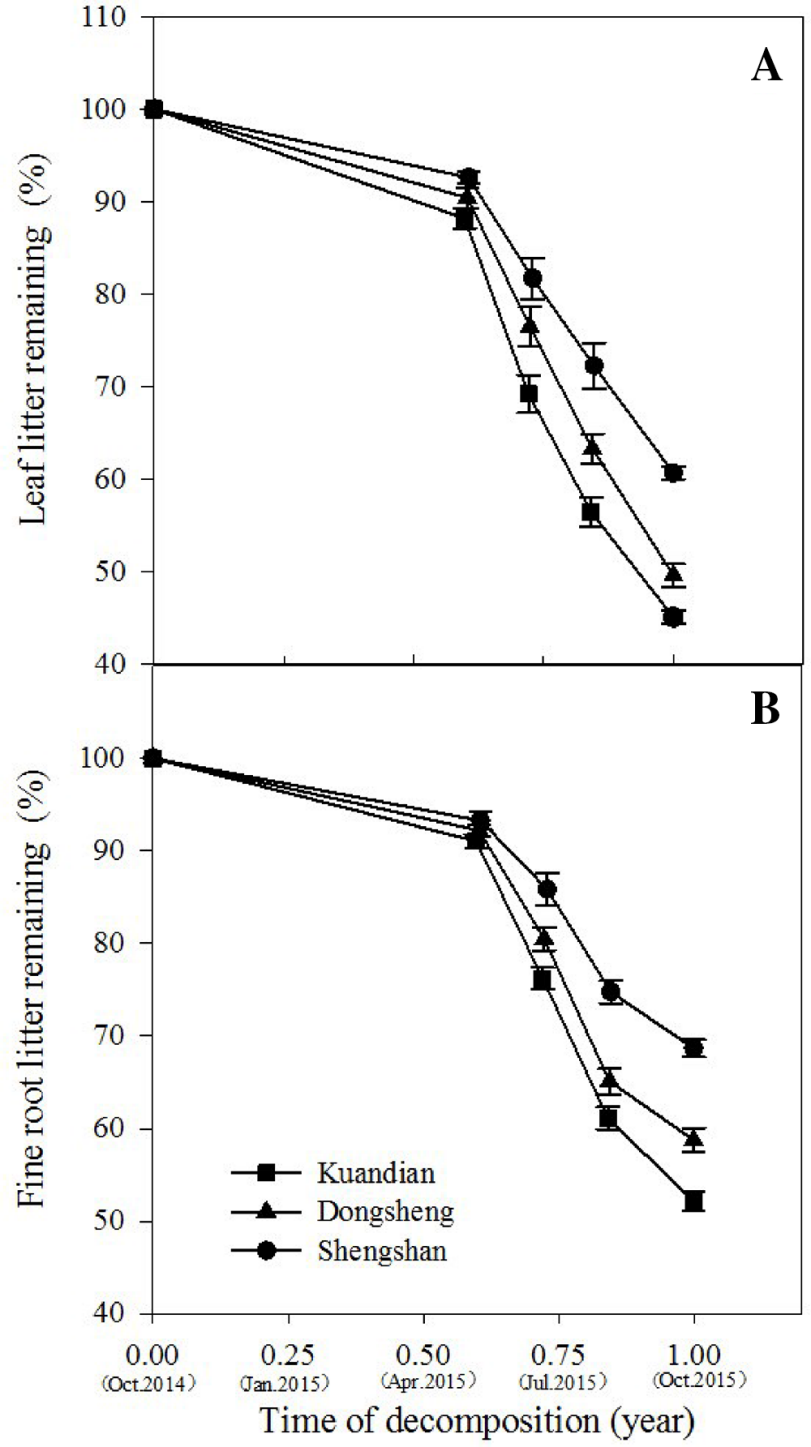
Leaf litter mass **(A)** and fine-root litter mass **(B)** remaining (% of initial) of three mixed coniferous–deciduous forests during 1-year period. Vertical bars indicate standard errors of means (n = 3).

**Figure 8:**
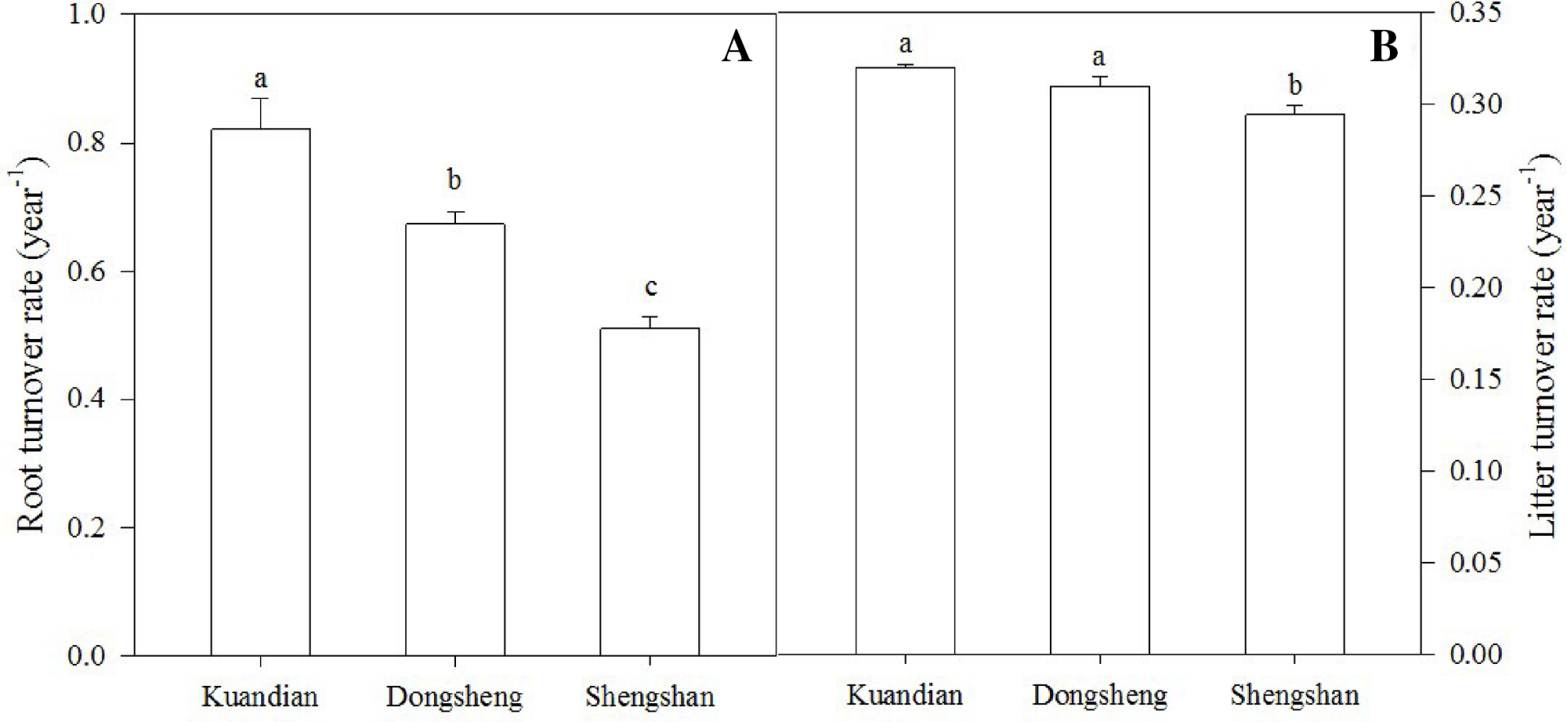
Fine-root turnover rate **(A)** and litter turnover rate **(B)** of three mixed coniferous–deciduous forests. Vertical bars indicate standard errors of means (n = 3). Different letters are significantly different (*P* < 0.05) according to the Duncan *post hoc* test.

After 1 year in the field, the least litter mass remained in the leaf litter and root litter bags in Kundian, followed by Dongshen and Shengshan (**Figure 7**). Nevertheless, the temporal pattern of decomposition was quite similar among the sites, with limited decomposition during the cold winter and then a rapid decrease in remaining mass between March and October. When estimating the turnover rates (**Table 4** and **Figure 8**), we observed a statistically significantly higher turnover rate for Kundian, followed by Dongshen and Shengshan, for both leaf litter and fine roots. The stepwise regression indicated that for all sites fine-root turnover rates were controlled by their C:N and C:P ratio, as well as by the SWC and the soil C:N ratio (**Table 5**). No microbial-related variables were significantly correlated to the turnover rate. For the leaf litter, turnover rate was controlled by its C:N ratio, but also by the soil C:N ratio and the F:B ratio. In addition, Korean pine basal area and tree density also partially controlled the leaf litter decay constant (**Table 5**).

**Table 4:** Mean annual decay constant (*k*) of leaf and fine-root litter of mixed coniferous–deciduous forests dominated by Korean pine in Kuandian, Dongsheng, and Shengshan. *R* ^2^, multiple coefficient of determination, and significance level of the exponential model (*P* < 0.05; *P* < 0.01) are also given. Values represent mean ± standard error (n = 3). Different letters within a soil layer are significantly different (*P* < 0.05) according to the Duncan *post hoc* test.

		k	*R* *^2^*
Leaf litter	Kuandian	0.751(0.147)a	0.783**
	Dongsheng	0.636(0.165)b	0.738**
	Shengshan	0.449(0.152)c	0.740**
			
Fine-root litter	Kuandian	0.619(0.061)a	0.873**
	Dongsheng	0.514(0.076)c	0.750**
	Shengshan	0.355(0.067)b	0.762**

** represents significant at P < 0.01.

**Table 5:** Results of the stepwise regression on annual decay constant.

Model		Unstandardized coefficients		Standardized coefficients	*t*	*P*
		*B*	Std. Error	β		
Fine-root k	(Constant)	1.098	0.073		15.097	0.000
	Root litter C:P	−0.001	0.000	−0.301	−6.339	0.003
	Soil C:N	−0.016	0.002	−0.310	−10.229	0.001
	Soil water content	0.002	0.000	0.294	5.859	0.004
	Root litter C:N	−0.003	0.001	−0.143	−3.132	0.035
Leaf litter k	(Constant)	1.685	0.013		127.315	0.000
	Leaf litter C:N	−0.013	0.001	−0.385	−19.975	0.000
	Soil C:N	−0.028	0.001	−0.481	−25.652	0.000
	Korean pine basal area	−0.004	0.000	−0.226	−31.918	0.000
	Tree density	−2.749E−5	0.000	−0.061	−8.106	0.004
	Fungi:bacteria	−0.899	0.205	−0.057	−4.383	0.022

## Discussion

Forest ecosystems, as a large and persistent carbon sink, play a more and more important role in the global carbon cycle under the context of climate change (Pan et al., 2011). Major carbon inputs into soils are fine root and leave litter, and it is necessary to analyse how they vary. Aboveground litterfall is the key ecosystem process for both nutrient and energy transfer from plant canopies to the soils. We observed that, for all study sites, the forest floor litter mass in fall was higher than in summer, as the litter of mixed deciduous–coniferous forests is mainly produced during fall when physiological activity is reduced (Richardson et al., 2010). The three sites had different litter and root production rates, inducing different litter stocks and fine-root biomass. This might be due to climatic factors, which are known to be major drivers of plant productivity (Beer et al., 2010), since the least productive site, Shengshan, was also the coldest and the driest. Nevertheless, it is difficult to clearly disentangle the effect since the sites also differed by their stand basal areas and their soil characteristics such as total SOC, STN, C:N, and C:P ratios. Nevertheless, litter mass is the result of a balance between litter inputs and decomposition, which we measured using the litter-bag method.

For leaf litter decomposition, the mass remaining after 1 year was in line with previous studies (Prescott et al., 2000; Parton et al., 2007). The main drivers of decomposition are related to climate and litter chemistry. We observed the fastest litter turnover rate at Kundian, which was also the site with the highest mean annual air temperature. Temperature indeed enhances microbial metabolic activity potentially leading to accelerated litter decomposition rate (Schindlbacher et al., 2011). C:N and C:P ratios of litter are generally higher than those of the microbial decomposers (Xu et al., 2013). Therefore, decomposers need to access exogenous nutrients to decompose the litter (Hobbie and Vitousek, 2000; Norris et al., 2013), explaining the significant effect of soil stoichiometry on litter decay rates. Moreover, other forest stand characteristics also appeared to be of major importance in our case (**Table 3**). Tree species composition effects on SOC stocks were already reported in a large-scale meta-analysis study (Vesterdal et al., 2013). This effect might be explained by an impact of the tree community and litter production on some soil characteristics driving the decomposition, such as moisture or temperature as observed by Villalobos-Vega et al. (2011) in neotropical savanna. Trees can also influence decomposition through the priming effect of their fresh litter inputs (Prévost-Bouré et al., 2010). Finally, decomposition rate is also partially controlled by soil microbial characteristics, in particular the microbial biomass and its F:B ratio (Salamanca et al., 2003). In our study, the slowest litter turnover rates were observed at Shengshan, where we also measured the lowest microbial biomass and the highest F:B ratio. Our F:B ratio values are in line with previous observations (Bååth and Anderson, 2003), and it is known that fungi play a major role in the first stages of litter decomposition (Voříšková and Baldrian, 2013). Therefore, the slowest decomposition and turnover rates observed at Shengshan are probably due to the lowest microbial biomass, although the highest F:B ratio was observed at Shengshan. Additionally, the F:B ratio in the three Korean pine forests decreased along the latitudinal gradient, reflecting that there is a shift in the function and composition of microbial communities among the three Korean pine forests along this latitudinal gradient that might be due to changes in the environmental conditions (e.g., temperature or moisture) or to changes in the stoichiometry of the litter.

Fine roots take an important part on the biogeochemical cycle of carbon in forest ecosystems, and up to 67% of the annual net primary production in forest ecosystem can be allocated to fine roots (Jackson et al., 1997; Janssens et al., 2002). Fine roots combine a short life span (< 1–9 years) (Matamala et al., 2003) and high decomposition rate (Silver and Miya, 2001). In our study, fine-root biomass profiles were in line with expectations (Riley et al., 2010), whereas their production was low compared to other studies (Finér et al., 2011). Measuring root productivity is difficult, and results are method dependent. Indeed, in-growth methods similar to what was applied in this study generally deliver lower productivity estimates than other methods (Finér et al., 2011). We speculate that this explains the rather low root productivity estimates in our study. Higher fine-root biomass was observed in Dongshen, compared to the other sites. Because the fine-root production rate was similar between Dongshen and Kuandian, the higher fine-root biomass at Dongshen was therefore probably due to the reduced mortality at that site, likely because of different physical conditions and nutrient availability. It is known that rapid fine-root turnover requires more nutrient and energy cost, such that at nutrient-limited sites root life span is typically longer, resulting in reduced turnover rates and lower efficiency of resource uptake (Schoettle et al., 1994).

Decay rates of fine roots were in line with previous observations (Gill and Jackson, 2000). In our study, decay rates depended primarily on the fine-root characteristics, and not on soil characteristics, with the exception of SWC and C:N ratio. The importance of C:N and C:P ratio of the decomposed material has been already underlined as a major driver of decomposition (Zhou et al., 2008). Interestingly, no microbial-related variables significantly affected the decomposition of fine roots in our study. This is in accordance with the work of (Wei et al. (2015), who observed that heterotrophic respiration was not controlled by microbial characteristics in subtropical forests.

In conclusion, in all study sites, the forest fine-root biomass and floor litter mass in fall were higher than in summer. The main drivers of decomposition were related to climate and litter chemistry, but also partially controlled by soil microbial characteristics, in particular the microbial biomass and its F:B ratio. The F:B ratio in the three Korean pine forests decreased along the latitudinal gradient, but we also observed that the fine-root and litter decomposition drivers were controlled by other environmental factors.

Finally, we showed that litter stocks (including aboveground and belowground litter) were different among the three study sites, even though they were all dominated by Korean pine. We further showed that the litter stock dynamics are the result of the balance between primary production and dead plant material decay. Both are controlled by stand characteristics and soil properties that may go into different directions. It is, nevertheless, difficult to clearly separate the effects of all the drivers. Manipulative experimental sites modifying, for instance, soil temperature or soil moisture are quite powerful tools to test ecological hypotheses (Genardzielinski et al., 2018), and we strongly advocate their use in future research, but also a combination with manipulated stand structure would be of great interest.

## Data Availability Statement

The datasets generated for this study are available on request to the corresponding author.

## Author Contributions

CX and BG formulated the original idea and developed methodology. FY, XS and CX performed sample processing. LL, FY and YW performed statistical analysis. CX, BG and IJ wrote the first draft of the manuscript.

## Funding

This study was financially supported by the National Natural Science Foundation of China (nos. 31370462 and 31770501).

## Conflict of Interest

The authors declare that the research was conducted in the absence of any commercial or financial relationships that could be construed as a potential conflict of interest.
